# Plasma levels of thrombomodulin, plasminogen activator inhibitor-1 and fibrinogen in elderly, diabetic patients with depressive symptoms

**DOI:** 10.1007/s40520-015-0504-3

**Published:** 2015-11-27

**Authors:** Malgorzata Gorska-Ciebiada, Malgorzata Saryusz-Wolska, Anna Borkowska, Maciej Ciebiada, Jerzy Loba

**Affiliations:** 1 Department of Internal Medicine and Diabetology, Medical University of Lodz, 251 Pomorska Street, 92-213 Lodz, Poland; 2 Department of General and Oncological Pneumology, Medical University of Lodz, 22 Kopcinskiego Street, 90-153 Lodz, Poland

**Keywords:** Depressive symptoms, Diabetes, Fibrinogen, PAI-1, Thrombomodulin

## Abstract

**Background:**

Diabetes, depression and aging have been associated with pro-inflammatory and prothrombotic state.

**Aim:**

The aim of the study was to determine the plasma levels of thrombomodulin, plasminogen activator inhibitor-1 (PAI-1) and fibrinogen in elderly diabetic patients with and without depressive symptoms and to examine factors (including thrombomodulin, PAI-1, fibrinogen levels) associated with depressive symptoms in elderly patients with type 2 diabetes (T2DM).

**Methods:**

A total of 276 T2DM elders were evaluated: 82 subjects with depressive symptoms and 194 controls. Data were collected concerning biochemical parameters and biomarkers.

**Results:**

Plasma thrombomodulin, PAI-1 and fibrinogen were elevated in patients with depressive symptoms compared to controls. Thrombomodulin level was correlated with fibrinogen and PAI-1 levels. All parameters were correlated with the Geriatric Depression Scale-30 score. The univariate logistic regression models revealed that variables which increased the likelihood of diagnosis of depressive symptoms in elderly patients with T2DM were: female sex, smoking habit, longer duration of T2DM, hyperlipidemia, neuropathy, increased number of co-morbidities, higher BMI, and higher levels of total and LDL cholesterol, thrombomodulin, PAI-1 and fibrinogen. In addition, the multivariable analysis indicated that female sex, smoking habit, increased number of co-morbidities, higher BMI, and higher levels of LDL cholesterol and thrombomodulin are the predisposing factors for depressive symptoms.

**Conclusions:**

Elderly diabetic patients with depressive symptoms have higher levels of thrombomodulin, PAI-1 and fibrinogen. Further prospective larger studies are needed to provide potential directions for the research, treatment and prevention of co-morbid depression and diabetes.

## Introduction

Depression is a disorder that affects a large proportion of the general population worldwide and is associated with functional impairment in normal life and at work, life style risk-factor interventions and poor compliance with medical therapy [1]. Depressive disorders are closely associated with type-2 diabetes (T2DM), obesity and metabolic syndrome [2, 3], and depression is related to increased medical morbidity and mortality in subjects with diabetes [4]. A higher prevalence of microvascular complications (retinopathy, nephropathy, neuropathy) and macrovascular complications has been noted among diabetic subjects with depression compared to those without [5].

A meta-analysis performed in the United States found the prevalence of depression among adult diabetic participants to range from 3.8 to 27.3 % [6]. Similarly high figures have also been reported by other studies based on general populations of seniors not only diabetics. The Cardiovascular Health Study found approximately 41 % of subjects to have a depressive mood [7] and a recent study by Gorska-Ciebiada et al. found 29.7 % of elderly patients with T2DM to have depressive symptoms [8]. Although many studies have confirmed the relationship between depression and diabetes, its pathogenesis is still not clear. Recent studies have shown that inflammation may be a common pathogenesis behind the accelerated development of diabetes and depression [9–11]. Several biological alterations have been reported in subjects with T2DM and depressive disorders, including activity of the hypothalamic–pituitary–adrenal axis leading to hypercortisolism, alterations in serotonin and norepinephrine metabolism, sympathetic nervous system activation, increases in C-reactive protein and inflammatory cytokines as tumor necrosis factor-alpha and interleukin-6 [12].

Diabetes, depression and aging have been associated with pro-inflammatory and prothrombotic state [13, 14]. Elevated levels of prothrombotic factors might account for some of the mechanisms which elevate the risk of cardiovascular disease in patients experiencing depression or diabetes. The relationship between depression and coagulation processes has rarely been investigated in the past. One large population study found depression to be positively associated with increased levels of prothrombotic factors such as PAI-1 (plasminogen activator inhibitor-1) and fVIII which may lead to hypercoagulability, and subsequent cardiovascular disease [15]. Other authors have observed elevated blood coagulation and fibrinolysis, D-dimer, PAI-1 and platelet activation in depressed patients [16, 17]. Another study reports that depressive symptoms were significantly associated with elevated levels of fibrinogen and C-reactive protein in a general population sample without overt cardiovascular disease [18]. Elevated levels of thrombomodulin have also been reported in participants with chronic diseases such as T2DM caused by inflammation and endothelial dysfunction [19]. As little literature data are available concerning coagulation factors in depressed subjects with diabetes, the aims of the study were twofold: First, evaluate levels of plasma thrombomodulin, PAI-1 and fibrinogen in elderly patients with T2DM with and without depressive symptoms and second, identify the factors (including thrombomodulin, PAI-1 and fibrinogen levels) associated with depressive symptoms in elderly patients with T2DM.

## Materials and methods

### Study population

A survey was conducted among 276 elders attending the outpatient clinic of the Department of Internal Medicine and Diabetology, University Hospital no 1, Lodz, Poland. A brief screening for recruitment was conducted by the investigators to identify potential participants. The inclusion criteria were an age of 65 and over, diagnosis of type 2 diabetes a minimum of 1 year earlier and the ability to understand and cooperate with study procedures. The exclusion criteria were a diagnosis of depression or dementia, use of possible or known cognition-impairing drugs in the previous 3 month, presence of a neoplasm, ongoing alcohol or substance abuse, severe visual, mobility, or motor coordination impairment, history of head trauma, inflammatory or infectious brain disease, severe neurological or psychiatric illness.

Written consent was obtained from the participants at the beginning of the study. The first part of the visit included a morning blood draw after a 10–12 h overnight fast, blood pressure measurements, height and weight assessment and complete physical examination. The patients then ate breakfast, and a capillary glucose level assessment was performed to ensure that participant was not hypoglycemic at the time of cognitive testing. The second part of the visit took place in a private area in the clinic. The subjects completed a questionnaire describing baseline demographics and underwent cognitive testing.

### Participant characteristics, clinical evaluation and risk factor assessment

Demographic variables and possible risk factors were recorded in a standardized interview. Weight and height were measured to calculate body mass index [BMI = weight/height^2^ (kg/m^2^)]. The systolic and diastolic blood pressures (mmHg) were measured with the patient in a sitting position after 5 min of rest. The detailed medical history of type 2 diabetes was taken: diabetes duration, current treatment for diabetes and complications if present, family history of diabetes, co-morbid diseases of the patient (hyperlipidemia, hypertension, cardiovascular disease, lung disease, cancer, gastrointestinal tract diseases) and their treatment. Educational level was recorded in years of education. Diabetic vascular complications were assessed based on the existence of nephropathy, retinopathy, neuropathy, cardiovascular disease (CVD) and stroke. Hypertension was defined as either a history of hypertension or use of any antihypertensive agents, Hyperlipidemia was defined as the use of any lipid lowering agent, or the presence of an untreated 2.6 mmol/l serum LDL cholesterol level and/or 1.7 mmol/l triglyceride level.

### Blood biochemistry

After overnight fasting, blood samples were taken by venipuncture to assess serum levels of glycosylated hemoglobin (HbA1c), total cholesterol, triglycerides, low-density lipoprotein cholesterol (LDL-C) and high-density lipoprotein cholesterol (HDL-C). All the parameters were measured in a centralized laboratory.

### Determination of plasma thrombomodulin, PAI-1 and fibrinogen

The plasma levels of thrombomodulin, PAI-1 and fibrinogen were assessed using ELISA kit (thrombomodulin: American diagnostica Inc, Stamford, CT, USA; PAI-1: R&D System, Minneapolis, USA; fibrinogen: EIAab, Wuhan, China) according to the instructions given by the manufacturer.

### Neuropsychological evaluations

All participants underwent the following tests: the Montreal Cognitive Assessment (MoCA) [20] to evaluate cognitive impairment, the long version of the Geriatric Depression Scale (GDS-30) [21] to assess the depressive symptoms, Katz Basic Activities of Daily living (BADL) and Lawton Instrumental Activities of Daily Living (IADL) questionnaires to collect information on daily activities [22, 23]. The MoCA tests 8 cognitive domains, visual–spatial ability, attention, executive function, immediate memory, delayed memory, language, abstraction, calculation, and orientation, for a maximum total score of 30. The normal MoCA score is ≥26, with one point added if the subject has fewer than 12 years of formal education. The MoCA is better than other tools to detect MCI in the elderly patients with type 2 diabetes [24]. MCI was diagnosed based on criteria established in the 2006 European Alzheimer’s Disease Consortium which constitute the currently available standard test [25, 26]. These criteria include absence of dementia. The cut-off points for MoCA scores (19/30) are recommended for the diagnosis of ‘dementia’ in epidemiological studies. Patients with a score of 19 and below were excluded from the study as experiencing dementia and sent to a psychiatrist for further care. The criteria mentioned above included also absence of major repercussions on daily life (in our study, measured by Katz BADL and Lawton IADL).

This interview was followed by an application of the Geriatric Depression Scale (GDS) for mood assessment [21]. The GDS consists of 30 items. Scores ranging from 0 to 9 are considered normal, 10–19 to indicate depressive symptoms, and scores 20 and above resulted in the subject being excluded from the study as demonstrating severe depressive symptomatology. Such subjects were referred to a psychiatrist for further diagnosis.

According to the criteria mentioned above, 276 older subjects with type 2 diabetes were selected into two groups: patients with depressive symptoms and controls (patients without depressive symptoms).

### Ethics

The study was operated in accordance with the World Medical Association’ Declaration of Helsinki and its later amendments. Each participant was assigned an identification number for privacy. The purpose, nature and potential risks of the experiments were fully explained to the subjects, and all subjects gave written, informed consent prior to their inclusion in the study. The study was approved by the independent local ethics committee of the Medical University of Lodz.

### Statistical analysis

All continuous results are presented as means ± SD. Frequencies and percentages were calculated to enable comparison of characteristics between patients with depressive symptoms and controls. Normality of distributions was assessed using the Shapiro–Wilk test. The descriptive statistics for the categorical variables were tested using the *χ*^2^, and the continuous variables using the Student’s *T* test or the Mann–Whitney *U* test whenever applicable. Relationships were assessed with Pearson’s correlation analysis for normally distributed variables and Spearman rank correlation for non-normally distributed variables. As many factors can influence the results, the simple logistic regression model was used to select so-called independent factors which increase the selection risk of depressive symptoms in elderly patients with type 2 diabetes. The independent variables entered in the model at step one were: demographic variables (age, gender, education), duration of diabetes, glycaemic control (HbA1c level), cardiovascular diseases (MI, angina, stroke), cardiovascular risk factors (BMI, smoking status, hyperlipidaemia, previous HA or use of HA drugs), microvascular complications, presence of mild cognitive impairment, number of co-morbid conditions, and the levels of total, LDL, HDL cholesterol, triglycerides, thrombomodulin, PAI-1 and fibrinogen. The multivariable regression model was used to select the “strongest” factor from the independent risk factors. All significant variables with *p* < 0.05 included in simple logistic regression model were introduced to this analysis. The multivariable model was optimized, using a stepwise approach (backward elimination with Wald criteria). Odds ratios (OR) were computed and presented with the 95 % interval of confidence (CI). A *p* value of less than 0.05 was considered statistically significant. Statistica 10.0 (StatSoft, Poland, Krakow) was used for analysis.

## Results

### General description of depressive subjects and controls

The demographic and clinical characteristics of the study group are presented in Tables 1 and 2. The results of the *χ*^2^ test indicated that male to female ratio varied significantly between patients with depressive symptoms and controls (*p* < 0.001). Patients with depressive symptoms were significantly more likely to be female, to have a smoking habit, to be diagnosed with hyperlipidemia, neuropathy and treated with insulin (test *χ*^2^). There were no significant differences between the groups with regard to the presence of CVD, stroke, hypertension, retinopathy, nephropathy or prevalence of mild cognitive impairments (Table 1). Furthermore, the Mann–Whitney *U* test and *T* test showed that patients with depressive symptoms had a longer duration of diabetes, higher number of co-morbidities, higher BMI and the level of total cholesterol and LDL cholesterol (Table 2). Lastly, no significant differences were found between the groups in age, years of education, systolic and diastolic blood pressure, the plasma levels of fasting glucose, or levels of HbA1c, triglycerides or HDL cholesterol (*p* > 0.05).

**Table 1:** Demographic and clinical characteristics of type 2 diabetic elderly patients

	Type 2 diabetes with depressive syndrome (*n* = 82)	Controls (*n* = 194)	*χ* ^2^	*p* value
Sex, male/female*	15/67	112/82	36.09	<0.001
Smoked tobacco regularly*	47 (57.3 %)	46 (23.7 %)	29.14	<0.001
Macrovascular complications previous CVD	33 (40.2 %)	76 (39.17 %)	0.03	0.86
Stroke	7 (8.53 %)	7 (3.6 %)	2.91	0.08
Previous HA/use of HA drugs	60 (73.17 %)	153 (78.86 %)	1.06	0.3
Hyperlipidemia*	76 (92.6 %)	142(73.2 %)	13.19	<0.001
Microvascular complications retinopathy	35 (42.6 %)	86 (44.32 %)	0.06	0.8
Nephropathy	27 (32.9 %)	70 (36.08 %)	0.25	0.61
Neuropathy*	33 (40.2 %)	23 (11.8 %)	28.7	<0.001
Treatment OAD	66 (80.4 %)	157 (80.9 %)	0.04	0.81
Insulin*	60 (73.2 %)	70 (36.0 %)	31.8	<0.001
Presence MCI, %	25 (30.4 %)	62 (31.9 %)	0.06	0.81
Other diseases				
Gastrointestinal tract disease	40 (48.7 %)	70 (36.1 %)	1.61	0.2
Thyroid disease	36 (43.9 %)	53 (27.3 %)	3.55	0.06
Kidney disease	21 (25.6 %)	45 (23.2 %)	0.11	0.74
Heart failure	20 (24.4 %)	38 (19.6 %)	0.51	0.47
Atrial fibrillation	21 (25.6 %)	36 (18.6 %)	1.12	0.28
Lung disease	12 (14.6 %)	25 (12.8 %)	0.12	0.73

*CVD* cardiovascular disease, *HA* hypertension, *OAD* oral anti-diabetic drug, *MCI* mild cognitive impairment

* Significance, *p* < 0.05; *χ* ^2^ test was used to test for significant differences between patients with depressive syndrome and those without depressive syndrome (controls)

**Table 2:** Characteristics and biochemical parameters of type 2 diabetic elderly patients

	Type 2 diabetes with depressive syndrome (*n* = 82)	Controls (*n* = 194)	*Z*/*t*	*p* value
Age (years)	74.4 ± 5.0	73.2 ± 4.7	1.68	0.09
Education-years	11.2 ± 2.4	11.3 ± 2.3	−0.27	0.78
Duration of T2DM (years)*	10.83 ± 6.72	7.78 ± 5.8	4.7	<0.001
BMI (kg/m^2^)*	32.03 ± 3.6	29.02 ± 3.32	6.07	<0.001
Thrombomodulin (ng/mL)*	6.03 ± 2.12	3.81 ± 0.84	7.78	<0.001
Fibrinogen (g/L)*	4.12 ± 1.71	2.69 ± 1.22	6.12	<0.001
PAI-1 (ng/mL)*	38.21 ± 8.47	31.32 ± 5.57	6.63	<0.001
HbA1c (%)	7.35 ± 0.77	7.19 ± 0.64	1.3	0.19
HbA1c (mmol/mol)	57 ± 0.77	55 ± 0.64	1.3	0.19
CHOL (mmol/L)*	11.59 ± 2.19	9.75 ± 1.93	6.67	<0.001
LDL (mmol/L)*	6.93 ± 1.83	5.69 ± 1.5	5.73	<0.001
TG (mmol/L)	9.82 ± 2.4	9.58 ± 2.16	0.13	0.89
HDL (mmol/L)	2.52 ± 0.47	2.57 ± 0.53	−0.12	0.90
Co-morbidity (*n*)*	6.43 ± 3.07	3.91 ± 2.81	6.28	<0.001
GDS score*	15.9 ± 2.8	3 ± 2.7	13.1	<0.001
Systolic blood pressure (mmHg)	135.36 ± 17.34	136.55 ± 15.4	−0.63	0.52
Diastolic blood pressure (mmHg)	74.43 ± 7.37	75.26 ± 8.11	−0.66	0.51
Fasting plasma glucose (mmol/L)	129.6 ± 27.8	129.2 ± 25.4	0.17	0.86

Data are mean − SD values

*DM* diabetes mellitus, *BMI* body mass index, *PAI-1* plasminogen activator inhibitor 1, *HbA1c* glycosylated hemoglobin, *CHOL* total cholesterol, *HDL* high-density lipoprotein cholesterol, *LDL* low density lipoprotein cholesterol, *TG* triglycerides, *GDS* geriatric depression scale

* Significance, *p* < 0.05; Mann–Whitney *U* test (*Z*), or *T* test (*t*) were used to test for significant differences between patients with depressive syndrome and those without depressive syndrome (controls)

### Thrombomodulin, PAI-1 and fibrinogen in depressive subjects and controls

Plasma thrombomodulin, PAI-1 and fibrinogen were significantly increased in patients with depressive symptoms compared to controls (*p* < 0.001) (Table 2). As expected, in the group of patients with depressive symptoms plasma thrombomodulin level was positively correlated with fibrinogen level (*r* = 0.79, *p* < 0.001) and with PAI-1 level (*r* = 0.76, *p* < 0.001). Fibrinogen level was also correlated with PAI-1 level (*r* = 0.75, *p* < 0.001). Furthermore, thrombomodulin, PAI-1 and fibrinogen concentrations were highly correlated with GDS-30 score. A positive but weak correlation was found between these parameters and total cholesterol and between thrombomodulin and LDL cholesterol level. The results indicated that thrombomodulin and fibrinogen concentrations were weakly correlated with HbA1c level. The results are presented in Table 3.

**Table 3:** Relationship of thrombomodulin, PAI-1 and fibrinogen with other clinical indicators in the elderly patients with T2DM and depressive syndrome

	Thrombomodulin, *r*	*p*	Fibrinogen, *r*	*p*	PAI-1, *r*	*p*
GDS score	0.63*	<0.001	0.54*	<0.001	0.58*	<0.001
HbA1c	0.32*	0.004	0.23*	0.03	0.16	0.14
CHOL (mmol/L)	0.45*	<0.001	0.45*	<0.001	0.32*	0.003
LDL (mmol/L)	0.23*	0.036	0.17	0.11	0.15	0.17
TG (mmol/L)	0.18	0.1	0.17	0.12	0.06	0.55
HDL (mmol/L)	−0.05	0.6	0.05	0.6	0.09	0.38
Thrombomodulin (ng/mL)	1					
Fibrinogen (g/L)	0.79*	<0.001	1			
PAI-1 (ng/mL)	0.76*	<0.001	0.75*	<0.001	1	

*BMI* body mass index, *PAI-1* plasminogen activator inhibitor 1, *HbA1c* glycosylated hemoglobin, *CHOL* total cholesterol, *HDL* high-density lipoprotein cholesterol, *LDL* low density lipoprotein cholesterol, *TG* triglycerides, *GDS* geriatric depression scale

* Significance, *p* < 0.05; *r* correlation coefficient

### Logistic regression models

The univariate logistic regression models revealed that variables which increased the likelihood of having been diagnosed with depressive symptoms in elderly patients with type 2 diabetes were female sex, smoking habit, longer duration of T2DM, hyperlipidemia, neuropathy, increased number of co-morbidities, higher BMI, and higher levels of total and LDL cholesterol, thrombomodulin, PAI-1 and fibrinogen (Table 4).

**Table 4:** Assessment results of the risk of having depressive syndrome in a simple logistic regression model in the elderly patients with T2DM

Variables analyzed	*β*	SE of β	*p* value	OR	95 % CI
Age (years)	0.05	0.02	0.054	1.05	0.99–1.11
Sex: female*	0.9	0.16	<0.001	2.47	1.8–3.38
Education (years)	−0.26	0.04	0.8	0.98	0.88–1.09
Smoked tobacco regularly*	0.73	0.14	<0.001	2.08	1.58–2.74
Duration of DM2 (years)*	0.08	0.02	0.001	1.07	1.03–1.13
Previous stroke	0.45	0.27	0.09	1.57	0.92–2.71
Previous CVD	0.14	0.02	0.86	1.02	0.78–1.33
Previous HA or use of HA drugs	0.15	0.05	0.3	1.17	0.86–1.57
Hyperlipidaemia*	0.76	0.22	0.001	2.15	1.38–3.36
Retinopathy	0.13	0.03	0.8	1.03	0.79–1.34
Nephropathy	0.14	0.07	0.6	1.07	0.8–1.41
Neuropathy*	0.8	0.15	<0.001	2.24	1.64–3.05
Co-morbidity (*n*)*	0.27	0.04	<0.001	1.3	1.19–1.43
Presence MCI	0.14	0.03	0.8	1.03	0.78–1.36
BMI (kg/m^2^)*	0.24	0.04	<0.001	1.27	1.17–1.37
HbA1c (%)	0.32	0.19	0.09	1.37	0.95–1.99
CHOL (mmol/L)*	0.02	0.004	<0.001	1.03	1.02–1.03
LDL (mmol/L)*	0.02	0.005	<0.001	1.03	1.02–1.03
TG (mmol/L)	0.03	0.003	0.39	1.0	0.99–1.0
HDL (mmol/L)	−0.01	0.009	0.53	0.99	0.96–1.02
Thrombomodulin (ng/mL)*	0.98	0.12	<0.001	2.66	2.09–3.39
Fibrinogen (g/L)*	0.65	0.1	<0.001	1.9	1.57–2.32
PAI-1 (ng/mL)*	0.14	0.02	<0.001	1.15	1.1–1.21

*β* regression coefficient, *CI* confidence interval for odds ratio, *OR* odds ratio, *SE* standard error, *DM* diabetes mellitus, *CVD* cardiovascular disease, *HA* hypertension, *MCI* mild cognitive impairment, *BMI* body mass index, *PAI-1* plasminogen activator inhibitor 1, *HbA1c* glycosylated hemoglobin, *CHOL* total cholesterol, *HDL* high-density lipoprotein cholesterol, *LDL* low density lipoprotein cholesterol, *TG* triglycerides

* Significance, *p* < 0.05

Table 5 shows the risk of depressive symptoms occurring based on multivariable regression. Female sex, smoking habit, increased number of co-morbidities, higher BMI, and higher levels of LDL cholesterol and thrombomodulin are the factors increasing the likelihood of having depressive symptoms in elderly patients with type 2 diabetes in multivariable model.

**Table 5:** Assessment results of the risk of having depressive syndrome in a multivariable logistic regression model in elderly patients with T2DM

Variables analyzed	*β*	SE of *β*	*p* value	OR	95 % CI
Sex: female*	0.95	0.22	<0.001	2.58	1.65–4.04
Smoked tobacco regularly*	0.63	0.2	0.002	1.89	1.26–2.82
Co-morbidity (n)*	0.16	0.06	0.01	1.17	1.04–1.33
BMI (kg/m^2^)*	0.12	0.05	0.034	1.13	1.0–1.26
LDL (mmol/L)*	0.014	0.006	0.025	1.014	1.0–1.02
Thrombomodulin (ng/mL)*	0.84	0.16	<0.001	2.35	1.7–3.19

*β* regression coefficient, *CI* confidence interval for odds ratio, *OR* odds ratio, *SE* standard error, *BMI* body mass index, *LDL* low density lipoprotein cholesterol

* Significance, *p* < 0.05

## Discussion

Higher levels of fibrinogen, thrombomodulin and PAI-1 were found in patients with depressive symptoms compared to those without. Elevated levels of coagulation factors in depressive elderly patients with T2DM could be a result of chronic low-grade inflammation and endothelial dysfunction. In recent years, some authors have proposed that inflammation may play a key role in the association between diabetes and depression. This hypothesis is based on the observation that higher concentrations of pro-inflammatory cytokines are involved in the pathogenesis of both T2DM and depressive symptoms independently [12, 27, 28]. The vascular endothelium is also involved in the regulation of hemostatic processes. Endothelial cells contribute to the generation of altered coagulation processes via increased expression of tissue factor, PAI-1, platelet activation and acute phase reactions that increase levels of coagulation factors such as fibrinogen. Cytokines stimulate the production of pro-thrombotic molecules such as PAI-1 and fibrinogen as an effect of the acute phase response. Current knowledge indicates that they are associated with the risk of DM complications [29].

Our findings indicate that plasma fibrinogen was significantly increased in patients with depressive symptoms compared to controls. Fibrinogen level is well known to be positively correlated with a higher risk of thrombosis as well as to be an indicator of acute inflammatory response [30]. Many studies have confirmed increased fibrinogen level in depressed patients and in diabetes [14, 18, 29]. A recently published work notes an association between elevated plasma fibrinogen levels and depression in 73,367 subjects [31]. High fibrinogen levels, reported in diabetic patients, result in the formation of denser and less permeable clots, more resistant to lysis, whereas elevated PAI-1 impairs fibrinolysis. There is also evidence that other components of the hemostasis system, such as PAI-1, could also be related to depression pathogenesis. A clinical study also reports that women with depression have higher PAI-1 levels than normal controls [15]. Similarly, depressed men have been reported to have higher levels of PAI-1 compared to non-depressed participants [32]. Consistent to other results, in our study elevated levels of PAI-1 were observed in depressed patients.

The endothelium also produces anticoagulant factors such as thrombomodulin. Thrombomodulin is an endothelial cell surface receptor for thrombin and functions as an anticoagulant through formation of a thrombin–thrombomodulin complex. This complex inhibits fibrin formation and platelet activation, accelerates protein C activation and then inhibits further formation of thrombin [33]. Subjects with chronic diseases related to inflammation and endothelial dysfunction, such as T2DM, have elevated levels of thrombomodulin [19]. Fujiwara et al. found also higher levels of thrombomodulin in type 2 diabetic patients with retinopathy than in those without, and they propose that this parameter could serve as sensitive indicator of endothelial damage [34]. A recently published general population-based study reports that endothelial dysfunction, as quantified by flow-mediated dilatation and circulating biomarkers (including thrombomodulin), was associated with a higher level of depressive symptoms. This relationship was independent of age, sex, diabetes, CVD risk factors, physical activity, dietary intake and education level. They concluded that endothelial dysfunction plays an important role in the pathophysiology of depression [35].

The present study is the first to demonstrate increased levels of thrombomodulin in elderly diabetic patients with depressive symptoms. It is possible that the high levels of this marker are accounted for by vascular co-morbidities or metabolic syndrome. Vascular pathology has previously been observed in patients with depressive symptoms [36] and another study observed that thrombomodulin levels significantly increased with elevated metabolic syndrome load [37]. Therefore, the components of metabolic syndrome (BMI, smoking status, hyperlipidemia, previous HA or use of HA drugs) or presence of CVD were included in the logistic regression model used in the present study, giving consistent results with other studies [38, 39]. Such factors as female sex, smoking habit, increased number of co-morbidities, higher BMI, and higher levels of LDL cholesterol and thrombomodulin increase the likelihood of depressive symptoms in elderly patients with type 2 diabetes. Some authors also indicated that depression is highly prevalent among diabetics and the risk of depression might be increased in the presence of other co-morbid conditions [39]. In our study, we noticed higher proportion of popular co-morbidities in subjects with depressive symptoms, but they were not statistically significant. However, we showed increased number of co-morbidities is an important variable in multivariate analysis. We also noticed that our depressed patients tended to be older in age, however, it was not statistically significant. Diabetes is a risk factor for depression in older people. Larger studies showed that major depression is less common in old age but that lighter symptoms such as minor depression are more common than in younger age groups [40]. Depressive symptoms have important consequences for elderly patients with diabetes and their families like impact on the management of diabetes, compliance with the prescribed treatment, glucose-level monitoring, education.

It is well established that patients with T2DM can be hypercoagulable, as they demonstrate increases in a number of coagulation markers such as fibrinogen, PAI-1 and vWF [29]. The present study also identifies a positive correlation between the plasma concentrations of thrombomodulin and PAI-1 and fibrinogen, suggesting that the plasma concentration of thrombomodulin represents hypercoagulability in diabetic patients.

Furthermore correlations were found between these parameters and total cholesterol and between thrombomodulin level and LDL cholesterol. These findings confirm those of other studies, which observe that hyperlipidemia may have a possible effect on the coagulation system [41, 42]. Some data suggest that serum lipids have a close relationship with thrombogenesis as evidenced by the presence of activated blood coagulations factors (F-Vll and F-X) in the extrinsic coagulation system and by elevated PAI-1 activity in fibrinolysis [41]. Recently hypercholesterolemia has been found to increase fibrinogen blood level in a mouse model lacking LDL receptors, and is crucial to determine hypercoagulation [43].

### Limitations

Although this study provides important insights into the pathophysiology of mood alterations in elderly diabetic patients; it does have limitations.

First, the study population was relatively small, and the findings should be interpreted with caution.

Second, the study was not designed as longitudinal investigation. Our findings indicate only an association between the coexistence of diabetes and depression and the biochemical alterations found in this study. Further study is required to investigate the precise mechanisms underlying hypercoagulability and coexisting depressive symptoms in diabetic patients.

Third, this investigation was limited to patients with diabetes, and therefore, an association between coagulation factors with other parameters in subjects without diabetes should also be assessed.

## Conclusions

In summary, elderly diabetic patients with depressive symptoms were found to have higher plasma levels of thrombomodulin, PAI-1 and fibrinogen compared to controls. The multivariable logistic regression models revealed that variables which increased the likelihood of having been diagnosed with depressive symptoms were: female sex, the presence of a smoking habit, increased number of co-morbidities, higher BMI, higher levels of LDL cholesterol and thrombomodulin. Various pathophysiological mechanisms like susceptibility to blood clotting, increased inflammation, oxidative stress and some neurodegenerative disorders may underlie co-morbid depression and diabetes. Therefore, further prospective larger studies are needed which can provide potential directions for research, treatment and prevention of these conditions.

## References

[1] DiMatteo MR, Lepper HS, Croghan TW (2000). Depression is a risk factor for noncompliance with medical treatment. Arch Intern Med.

[2] Shelton RC, Miller AH (2010). Eating ourselves to death (and despair): the contribution of adiposity and inflammation to depression. Prog Neurobiol.

[3] Bouwman V, Adriaanse MC, Riet E, Snoek FJ, Dekker JM, Nijpels G (2010). Depression, anxiety and glucose metabolism in the general dutch population: the new Hoorn study. PLoS One.

[4] Zhang X, Norris SL, Gregg EW, Cheng YJ, Beckles G, Kahn HS (2005). Depressive symptoms and mortality among persons with and without diabetes. Am J Epidemiol.

[5] de Groot M, Anderson R, Freedland KE, Clouse RE, Lustman PJ (2001). Association of depression and diabetes complications: a meta-analysis. Psychosom Med.

[6] Anderson RJ, Freedland KE, Clouse RE, Lustman PJ (2001). The prevalence of comorbid depression in adults with diabetes: a meta-analysis. Diabetes Care.

[7] Barnes DE, Alexopoulos GS, Lopez OL, Williamson JD, Yaffe K (2006). Depressive symptoms, vascular disease, and mild cognitive impairment findings from the Cardiovascular Health Study. Arch Gen Psychiatry.

[8] Gorska-Ciebiada M, Saryusz-Wolska M, Ciebiada M, Loba J (2014). Mild cognitive impairment and depressive symptoms in elderly patients with diabetes- prevalence, risk factors and co-morbidity. J Diabetes Res.

[9] Pizzi C, Manzoli L, Mancini S, Bedetti G, Fontana F, Costa GM (2010). Autonomic nervous system, inflammation and preclinical carotid atherosclerosis in depressed subjects with coronary risk factors. Atherosclerosis.

[10] Howren MB, Lamkin DM, Suls J (2009). Associations of depression with C-reactive protein, IL-1, and IL-6: a meta-analysis. Psychosom Med.

[11] Gorska-Ciebiada M, Saryusz-Wolska M, Borkowska A, Ciebiada M, Loba J (2015). Serum levels of inflammatory markers in depressed elderly patients with diabetes and mild cognitive impairment. PLoS One.

[12] Stuart MJ, Baune BT (2012). Depression and type 2 diabetes, inflammatory mechanisms of a psychoneuroendocrine co-morbidity. Neurosci Biobehav Rev.

[13] Kim HK, Kim JE, Park SH, Kim YI, Nam-Goong IS, Kim ES (2014). High coagulation factor levels and low protein C levels contribute to enhanced thrombin generation in patients with diabetes who do not have macrovascular complications. J Diabetes Complications.

[14] Kop WJ, Gottdiener JS, Tangen CM, Fried LP, McBurnie MA, Walston J, Newman A, Hirsch C, Tracy RP (2002). Inflammation and coagulation factors in persons >65 years of age with symptoms of depression but without evidence of myocardial ischemia. Am J Cardiol.

[15] Eskandari F, Mistry S, Martinez PE, Torvik S, Kotila C, Sebring N, Drinkard BE, Levy C, Reynolds JC, Csako G, Gold PW, Horne M, Cizza G, POWER (Premenopausal, Odteopenia/Osteoporosis, Women, Alendronate, Depression) Study Group (2005). Younger, premenopausal women with major depressive disorder have more abdominal fat and increased serum levels of prothrombotic factors: implications for greater cardiovascular risk. Metabolism.

[16] Geiser F, Meier C, Wegener I, Imbierowicz K, Conrad R, Liedtke R, Oldenburg J, Harbrecht U (2008). Association between anxiety and factors of coagulation and fibrinolysis. Psychother Psychosom.

[17] Nemeroff CB, Musselman DL (2000). Are platelets the link between depression and ischemic heart disease?. Am Heart J.

[18] Panagiotakos DB, Pitsavos C, Chrysohoou C, Tsetsekou E, Papageorgiou C, Christodoulou G, Stefanadis C, ATTICA study (2004). Inflammation, coagulation, and depressive symptomatology in cardiovascular disease-free people; the ATTICA study. Eur Heart J.

[19] Aso Y, Inukai T, Takemura Y (1998). Mechanisms of elevation of serum and urinary concentrations of soluble thrombomodulin in diabetic patients: possible application as a marker for vascular endothelial injury. Metabolism.

[20] Nasreddine ZS, Phillips NA, B´edirian V, Charbonneau S, Whitehead V, Collin I, Cummings JL, Chertkow H (2005). The montreal cognitive assessment, MoCA: a brief screening tool for mild cognitive impairment. J Am Geriatr Soc.

[21] Yesavage J, Brink T, Rose T, Lum O, Huang V, Adev M, Leirer VO (1982). Development and validation of a geriatric depression screening scale: a preliminary report. J Psychiatr Res.

[22] Katz S, Downs TD, Cash HR, Grotz RC (1970). Progress in development of the index of ADL. Gerontologist.

[23] Lawton MP, Brody EM (1969). Assessment of older people: self-maintaining and instrumental activities of daily living. Gerontologist.

[24] Alagiakrishnan K, Zhao N, Mereu L, Senior P, Senthilselvan A (2013). Montreal cognitive assessment is superior to standardized mini-mental status exam in detecting mild cognitive impairment in the middle-aged and elderly patients with type 2 diabetes mellitus. Biomed Res Int.

[25] Petersen RC (2004). Mild cognitive impairment as a diagnostic entity. J Intern Med.

[26] Portet F, Ousset PJ, Visser PJ, Frisoni GB, Nobili F, Scheltens P, Vellas B, Touchon J, MCI Working Group of the European Consortium on Alzheimer’s Disease (EADC) (2006). Mild cognitive impairment (MCI) in medical practice: a critical review of the concept and new diagnostic procedure. Report of the MCI Working Group of the European Consortium on Alzheimer’s Disease. J Neurol Neurosurg Psychiatry.

[27] Doyle TA, de Groot M, Harris T, Schwartz F, Strotmeyer ES, Johnson KC, Kanaya A (2013). Diabetes, depressive symptoms, and inflammation in older adults: results from the health, aging, and body composition study. J Psychosom Res.

[28] Kampoli AM, Tousoulis D, Briasoulis A, Latsios G, Papageorgiou N, Stefanadis C (2011). Potential pathogenic inflammatory mechanisms of endothelial dysfunction induced by type 2 diabetes mellitus. Curr Pharm Des.

[29] Tousoulis D, Papageorgiou N, Androulakis E, Siasos G, Latsios G, Tentolouris K, Stefanadis C (2013). Diabetes mellitus-associated vascular impairment novel circulating biomarkers and therapeutic approaches. J Am Coll Cardiol.

[30] Best LG, North KE, Li X, Palmieri V, Umans JG, MacCluer J, Laston S, Haack K, Goring H, Diego VP, Almasy L, Lee ET, Tracy RP, Cole S (2008). Linkage study of fibrinogen levels: the strong heart family study. BMC Med Genet.

[31] Wium-Andersen MK, Orsted DD, Nordestgaard BG (2012). Association between elevated plasma fibrinogen and psychological distress, and depression in 73,367 individuals from the general population. Mol Psychiatry.

[32] Lahlou-Laforet K, Alhenc-Gelas M, Pornin M, Bydlowski S, Seigneur E, Benetos A, Kierzin JM, Scarabin PY, Ducimetiere P, Aiach M, Guize L, Consoli SM (2006). Relation of depressive mood to plasminogen activator inhibitor, tissue plasminogen activator, and fibrinogen levels in patients with versus without coronary heart disease. Am J Cardiol.

[33] Sadler JE (1997). Thrombomodulin structure and function. Thromb Haemost.

[34] Fujiwara Y, Tagami S, Kawakami Y (1998). Circulating thrombomodulin and hematological alterations in type 2 diabetic patients with retinopathy. J Atheroscler Thromb.

[35] van Sloten TT, Schram MT, Adriaanse MC, Dekker JM, Nijpels G, Teerlink T, Scheffer PG, Pouwer F, Schalkwijk CG, Stehouwer CD, Henry RM (2014). Endothelial dysfunction is associated with a greater depressive symptom score in a general elderly population: the Hoorn Study. Psychol Med.

[36] Camus V, Kraehenbühl H, Preisig M, Büla CJ, Waeber G (2004). Geriatric depression and vascular diseases: what are the links?. J Affect Disord.

[37] Alizadeh Dehnavi R, Beishuizen ED, van de Ree MA, Le Cessie S, Huisman MV, Kluft C, Princen HM, Tamsma JT (2008). The impact of metabolic syndrome and CRP on vascular phenotype in type 2 diabetes mellitus. Eur J Intern Med.

[38] Pizzi C, Manzoli L, Mancini S, Costa GM (2008) Analysis of potential predictors of depression among coronary heart disease risk factors including heart rate variability, markers of inflammation, and endothelial function. Eur Heart J 29:1110–1117 10.1093/eurheartj/ehn137 18400765

[39] Sweileh WM, Abu-Hadeed HM, Al-Jabi SW, Zyoud SH (2014). Prevalence of depression among people with type 2 diabetes mellitus: a cross sectional study in Palestine. BMC Public Health.

[40] Beekman ATF, Geerling SW, Deeg DJH, Smit JH, Schoevers RS, de Beurs E, Braam AW, Penninx BJWH, van Tilburg W (2002). The natural history of late-life depression: a 6-year prospective study in the community. Arch Gen Psychiatr.

[41] Ohni M, Mishima K, Nakajima K, Yamamoto M, Hata Y (1995). Serum triglycerides and blood coagulation factors VII and X, and plasminogen activator inhibitor-1. J Atheroscler Thromb.

[42] Morishita E, Jokaji H, Matsuda T (1995). Hyperlipidemia and hemostatic system. J Atheroscler Thromb.

[43] Evangelho JS, Casali KR, Campos C, De Angelis K, Veiga AB, Rigatto K (2011). Hypercholesterolemia magnitude increases sympathetic modulation and coagulation in LDLr knockout mice. Auton Neurosci.

